# Development of Neutralizing Antibodies against Zika Virus Based on Its Envelope Protein Structure

**DOI:** 10.1007/s12250-019-00093-5

**Published:** 2019-04-24

**Authors:** Chunpeng Yang, Rui Gong, Natalia de Val

**Affiliations:** 1 CAS Key Laboratory of Special Pathogens and Biosafety, Wuhan Institute of Virology, Chinese Academy of Sciences, Wuhan, 430071 China; 2 University of Chinese Academy of Sciences, Beijing, 100049 China; 3 Center for Molecular Microscopy, Center for Cancer Research, National Cancer Institute, National Institutes of Health, Bethesda, MD 20892 USA; 4 Cancer Research Technology Program, Frederick National Laboratory for Cancer Research, Leidos Biomedical Research Inc, Frederick, MD 21701 USA

**Keywords:** Zika virus (ZIKV), Envelope protein, Neutralizing antibody, X-ray crystallography, Cryo-electron microscopy (cryo-EM)

## Abstract

As we know more about Zika virus (ZIKV), as well as its linkage to birth defects (microcephaly) and autoimmune neurological syndromes, we realize the importance of developing an efficient vaccine against it. Zika virus disease has affected many countries and is becoming a major public health concern. To deal with the infection of ZIKV, plenty of experiments have been done on selection of neutralizing antibodies that can target the envelope (E) protein on the surface of the virion. However, the existence of antibody-dependent enhancement (ADE) effect might limit the use of them as therapeutic candidates. In this review, we classify the neutralizing antibodies against ZIKV based on the epitopes and summarize the resolved structural information on antibody/antigen complex from X-ray crystallography and cryo-electron microscopy (cryo-EM), which might be useful for further development of potent neutralizing antibodies and vaccines toward clinical use.

## Introduction

Zika virus (ZIKV) is an arbovirus and it can be transmitted to humans by Aedes mosquitoes as well as by sexual interactions. As a member of the *Flaviviridae* family of positive strand RNA, ZIKV is close to some important human pathogens such as dengue virus (DENV), yellow fever virus (YFV), west nile virus (WNV), Japanese encephalitis virus (JEV), and tick-borne encephalitis virus (TBEV) (Wang* et al.*^2017^). Among these flaviviruses, DENV is the closest one to ZIKV. Some studies have proved that neutralizing antibodies isolated from convalescent patients infected by DENV or ZIKV showed cross-neutralizing ability (Barba-Spaeth* et al. *2016; Wang * et al.*^2016^). During the infection of the virus, humoral immune response plays an important role in the clearance of this invader. Antibodies execute their protective effects by virus neutralization or Fc-mediated effector functions (e.g., ADCC and CDC) (Lu* et al. *2018). For ZIKV infection, the specific monoclonal antibodies (mAbs) can neutralize the virus by strongly binding to the E proteins on the surface of the viral particle, and blocking the viral attachment to the host cell or by preventing the rearrangement of the E proteins before the membrane fusion step (Munjal* et al. *2017). In recent reports, lots of potent and specific/cross-reactive mAbs were isolated from convalescent patients and immunized animals (Stettler* et al.*^2016^; Wang* et al.*^2016^; Zhao* et al. *2016; Robbiani* et al.*^2017^). To better understand the mechanism of protection, several structures of E protein or virion in complex with neutralizing mAbs were resolved by X-ray crystallography and cryo-electron microscopy (cryo-EM), and different epitopes on E proteins were revealed. The information obtained from the structural analysis helps us understand the relationship between epitopes and neutralizing antibodies, which will facilitate the development of safer and more effective therapeutic antibodies and vaccines against infection by ZIKV as well as other flaviviruses.

## ZIKV Envelope Protein, an Ideal Target for Neutralizing mAbs

**Fig. 1:**
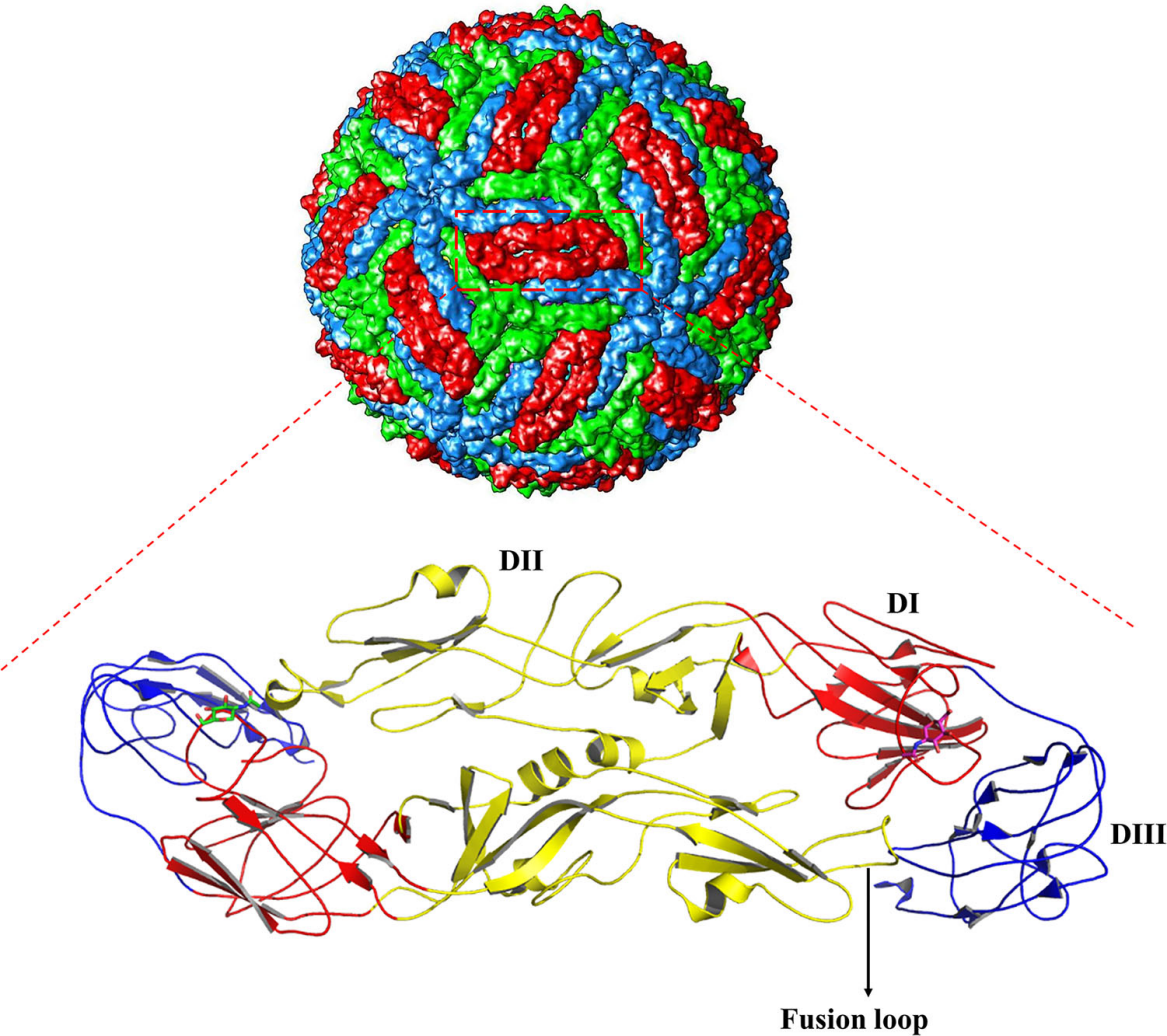
The Cα backbone of the E and M proteins in the icosahedral ZIKV particle showing the herringbone organization. The zoom part shows E protein dimer shown in ribbon form viewed down the two-fold axis. The color code fits the standard designation of E protein domains I (red), domain II (yellow) and domain III (blue). The PDB number of ZIKV virion is 5IZ7.

High-resolution structures of mature ZIKV were resolved by cryo-EM and the structural information indicated that the overall ZIKV structure is similar to those of other flaviviruses (Kostyuchenko* et al.*^2016^; Sirohi* et al. *2016). ZIKV is composed of 180 copies of E protein and forms a compact particle with icosahedral symmetry (Fig. 1). In detail, each copy of E protein contains three distinct domains in its ectodomain, named DI, DII and DIII. Domain I (DI) contains the N-terminus of E protein, domain II (DII) is an extended finger-like structure that includes the dimerization domain and also a pH-sensitive fusion loop that mediates viral fusion. The domain III (DIII) is an immunoglobulin-like domain that mediates attachment to target cells (Robbiani* et al. *2017). These three domains are connected to the viral membrane by two helices called stem anchor. The DI, DII and DIII are arranged so as to place DI in the center with DII and DIII flanking the two sides to form a monomer. The E monomer interacts with adjacent monomer in an antiparallel way to form a dimer (Fig. 1). Three E-dimers lay parallel to each other and form a structural unit known as a “raft” (Kostyuchenko* et al.*^2016^; Sirohi* et al. *2016; Sevvana* et al.*^2018^).

To entry the host cell, the E protein needs to interact with its receptor on the target cell (Hasan* et al.*^2018^). To date, although no specific receptor appears to be involved in the interaction of ZIKV with the host cell, studies from DENV and other flaviviruses show that E protein can bind many cellular factors, such as C-type lectin receptors, laminin receptor, T cell immunoglobulin and mucin domain (TIM) and TYRO3, AXL and MER (TAM) receptors, and integrin αvβ3 (Perera-Lecoin* et al.*^2013^; Sirohi and Kuhn 2017). After binding to these possible receptors, the virions undergo the low pH-dependent endocytosis and conformational rearrangement of E proteins, that happens in the endosomes. Then the viral membrane fuses with the host membrane and the RNA genome of the virus is released into the cytoplasm (Stiasny and Heinz 2006; Harrison 2008; Gerold* et al.*^2017^). However, the detailed process of the entry of the flavivirus into the host cell has not been fully understood and it still needs further studies.

Given the important function of the envelope protein in the life cycle of the flaviviruses, this is an ideal target for the development of neutralizing mAbs. Different studies have proved that each domain of E protein can elicit humoral immune response *in vivo* (Fibriansah and Lok 2016; Zhao* et al. *2016; Wu* et al.*^2017^; Qu* et al.*^2018^; Tai* et al.*^2018^). The neutralizing mAbs targeting ZIKV E protein were isolated from convalescent patients, immunized animals and phage display library (Deng * et al.*^2011^; Dejnirattisai* et al.*^2015^; Stettler* et al.*^2016^; Wang *et al*. 2016; Zhao* et al. *2016; Wu* et al.*^2017^). To better understand the epitopes information of those neutralizing mAbs in complex with ZIKV E protein, we will discuss more details in the following sections.

## Antibody-dependent Enhancement (ADE) of ZIKV

In the development of anti-ZIKV agents, the ADE phenomenon is very worrisome. This phenomenon initially explains that during DENV pathogenesis more severe symptoms will occur upon secondary infection by a heterologous DENV serotype. During this ADE phenomenon, cells bearing FcR can uptake and internalize antibody-coated viruses and further be infected (Dowd and Pierson 2011). Why don’t the mAbs neutralize the viruses but enhance the infection? This question can be explained by several factors: (1) The neutralizing activity of these mAbs is too weak to neutralize the viruses. (2) The blocking sites of E proteins do not affect the viral infection ability. (3) The concentration of these neutralizing mAbs is too low to neutralize the viruses. In addition, the existence of antigenic cross-reactivity is necessary for inducing ADE. Although the sequences of ZIKV and DENV E proteins are different, their structures are very similar. Different studies showed that antibodies isolated from DENV or ZIKV infected patients have high cross-neutralizing or cross-protective effects *in vitro* and *in vivo* (Barba-Spaeth* et al.*^2016^; Wang* et al. *2016). The *in vitro* assays have shown that this ADE phenomenon occurred between ZIKV and DENV (Li* et al.*^2017^) could increase the difficulty to develop antibody-based therapies against ZIKV or DENV infection.

Although a lot of work have been done to minimize the ADE, it is difficult to avoid it completely without any engineering of the mAbs. Currently, some research groups are doing some engineering to mutate the wild type Fc to “LALA” Fc and to eliminate the possible ADE (Sapparapu* et al. *2016; Stettler* et al. *2016; Fernandez* et al.*^2017^). Moreover, another study (Li* et al.*^2017^) has proved that neutralization and enhancement are two linked properties of the same antibody. This is consistent with “occupancy theory” that is used to describe the interaction of antibody and virus (Della-Porta and Westaway 1978).

## Neutralizing Antibodies Targeting Different Epitopes on ZIKV E Protein

As mentioned above, it has been found that all three domains of ZIKV E protein can be targeted by neutralizing antibodies. Here we classify these antibodies according to the binding epitopes based on the known structures.

## DI/DII Epitope

**Fig. 2:**
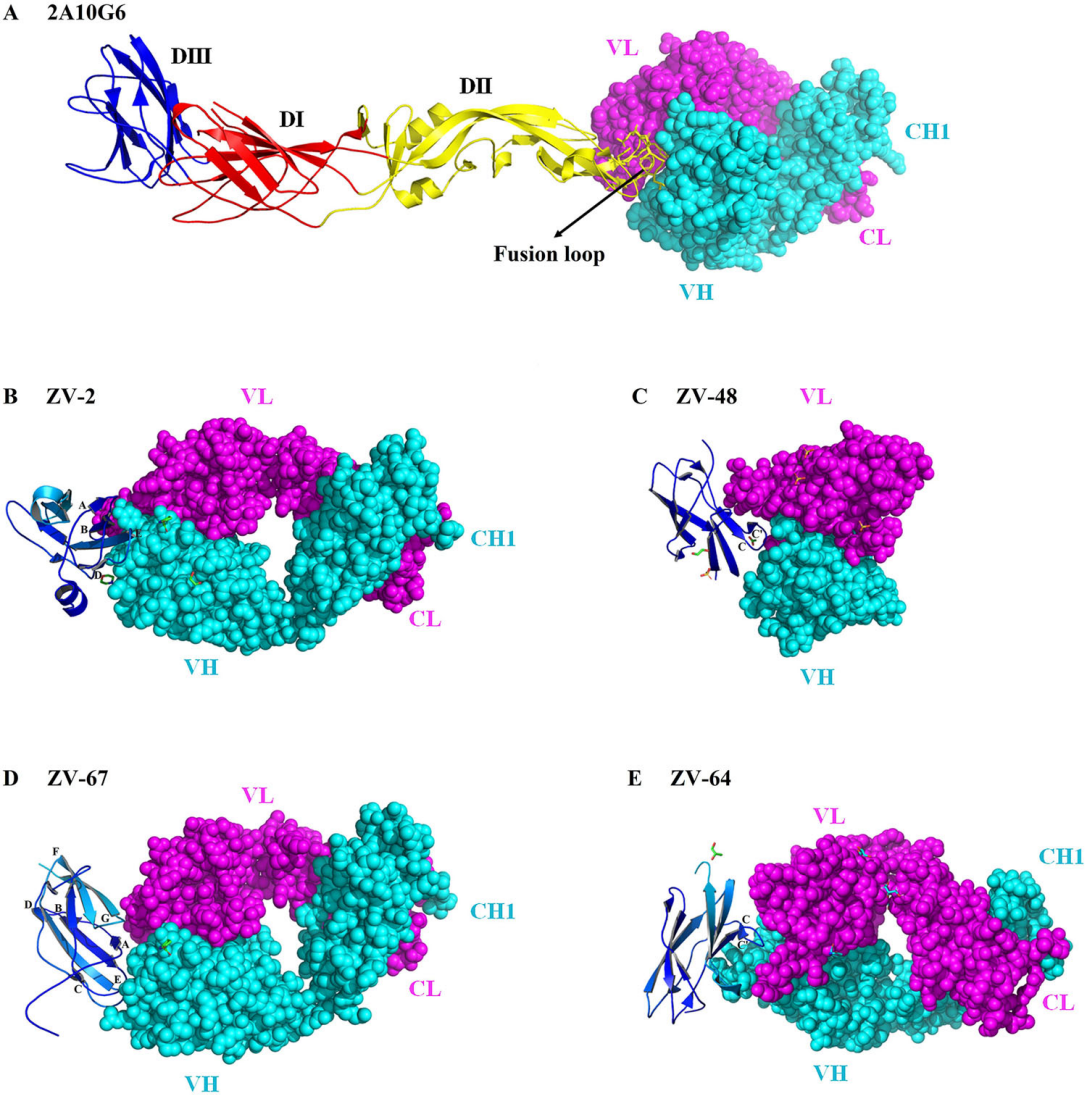
**A** The 2A10G6 Fab binds to the domain II tip of ZIKV-E monomer at a perpendicular angle with the fusion loop. **B** The ZV-2 Fab binds to the ABDE sheet of domain III of ZIKV E. **C** The ZV-48 binds to the C-C′ loop of domain III of ZIKV E. **D** The ZV-67 binds to the LR epitope of domain III of ZIKV E. **E** The ZV-64 binds to the C-C′ loop of domain III of ZIKV E.

There is an important target in DI/DII of ZIKV E protein called fusion loop (FL), which plays a key role in the fusion of viral membrane and host cell membrane. The first reported antibody targeting this FL was 2A10G6 (Deng* et al.*^2011^; Dai* et al.*^2016^), which was a broadly neutralizing mAb derived from immunized mice with inactivated DENV2. The structure of ZIKV E protein in complex with 2A10G6 Fab was resolved using X-ray crystallography (PDB: 5JHL). This structure revealed a highly conserved peptide ^98^DRXW^101^ within this fusion loop (Dai* et al. *2016) (Fig. 2A). Although it has been proved that 2A10G6 can neutralize ZIKV, its efficacy is weaker than that on DENV and WNV (Deng* et al.*^2011^; Dai *et al.*^2016^). This antibody targeting the fusion loop blocks the insertion of E protein into the endosomal membrane. According to the structure of viral particle, ZIKV displays higher thermal stability than other flaviviruses, which means less “breathing” of E proteins on the surface of the viral particle making the access by neutralizing mAbs to this fusion loop more complicated.

Although some studies showed that the FL is a dominant epitope to cause B cell response *in vivo*, the mAbs against this fusion loop are less potent than other mAbs focusing on different epitopes. In addition, the mAbs targeting this fusion loop might cause more severe viral infection via ADE (Dejnirattisai *et al*. 2010). Therefore, due to this ADE phenomenon, the use of mAbs targeting the fusion loop might not be highly recommended for the development of a future antiviral agent.

## DIII Epitope

The DIII of ZIKV E protein is an immunoglobulin-like domain and it primarily induces specific neutralizing antibodies *in vivo*. Several ZIKV DIII-specific neutralizing mAbs have been isolated from convalescent patients and immunized mice. Three different epitopes were identified in the DIII domain: (1) the lateral ridge (LR), (2) the C–C′ loop, and (3) the ABDE sheet regions. Corresponding to these three epitopes, four high-resolution X-ray structures were resolved: ZV-2 (ABDE sheet; PDB: 5KVD) (Fig. 2B), ZV-48 (C–C′ loop; PDB: 5KVE) (Fig. 2C), ZV-64 (C–C′ loop; PDB: 5KVF) and ZV-67 (LR; PDB: 5KVG) (Fig. 2D, 2E). However, only LR is a full exposed epitope on the mature virion, and both ABDE sheet and C–C′ loops are less accessible epitopes (Zhao* et al.*^2016^). Further studies showed that only mAbs targeting the LR loop were able to neutralize all four strains of ZIKV *in vitro* and provide protection in ZIKV lethal challenge models *in vivo*.

A recent study (Robbiani *et al.*^2017^) showed that DIII-LR epitope is also a protective epitope in ZIKV infected human. Sera from more than 400 donors from ZIKV epidemic areas of Mexico and Brazil were used to select high neutralizers. Two neutralizing mAbs (Z004 and Z006) derived from germline VH3-23/VK1-5 were isolated. These mAbs can provide protection against ZIKV and DENV1. Structures of Z006 (PDB: 5VIG) and Z004 (PDB: 5VIC) in complex with ZIKV and DENV1 DIII domains were obtained respectively and they show a similar mode of antigen recognition. Therefore, it is possible to engineer the DIII to mask the epitopes that fail to elicit neutralizing mAbs and get epitope-focused vaccine to induce more protective responses *in vivo*.

## Higher-order Structural Epitopes

Recently, a new class of highly potent neutralizing mAbs was isolated from DENV convalescent patients. Epitope mapping studies indicated that these mAbs can only recognize the epitopes on correctly formed E dimers. And thus the related epitopes were named E-dimer-dependent epitopes (EDE). In general, these mAbs lock the E dimer in pre-fusion and inhibit the transformation of dimer to trimer, which is the critical step for membrane fusion. Cross-neutralizing activity of these mAbs to ZIKV was further confirmed by other research groups (Dejnirattisai* et al.*^2015^; Barba-Spaeth* et al.*^2016^). There were two subsets of EDE mAbs (EDE1 mAbs and EDE2 mAbs), characterized by a differential requirement for glycosylation on the 150 loop for binding. The EDE1 mAbs bind better in the absence of glycan, whereas EDE2 mAbs bind better when the glycan is present (Barba-Spaeth et al. 2016). These EDE mAbs are able to neutralize ZIKV as potently as they neutralize DENV.

The complex structures of EDE1 C8/ZIKV E protein and EDE2 A11/ZIKV E protein were resolved (Barba-Spaeth* et al.*^2016^). For EDE1 C8 in complex with ZIKV E (PDB: 5LBS), the main cluster of interactions is centered on β-strand *b* of domain II and the fusion loop main chain. The absence of the N67 glycan in ZIKV showed that these contacts were not essential for binding. Compared to C8, the binding of EDE2 A11 extremely relies on the variable 150 loop in which glycosylation is not always present. This is clearly a drawback of EDE2 mAbs as demonstrated by their poor affinity and their strong induction of ADE. Because of these reasons, the epitopes of EDE1 mAbs are more suitable than that of EDE2 mAbs for development of the potent epitope-focused vaccines for ZIKV and DENV super serogroup (Barba-Spaeth* et al.*^2016^).

Different from the cross-neutralizing EDE mAbs, a ZIKV-specific mAb named ZIKV-117 was isolated from a ZIKV-infected patient. This ZIKV-117 was demonstrated to possess therapeutic potential, with no cross-reactivity to other flaviviruses (Sapparapu* et al.*^2016^; Hasan* et al.*^2017^). This ZIKV-117 was able to neutralize the virus by cross-linking the E monomers within dimers and also cross-linking neighboring dimers in the viral glycoprotein shell (Sapparapu* et al.*^2016^; Hasan* et al.*^2017^). Due to the existence of steric hindrance, saturation is achieved by binding of only 60 ZIKV-117 Fabs to the 180 chemically equivalent sites on the ZIKV (PDB: 5UHY).

**Fig. 3:**
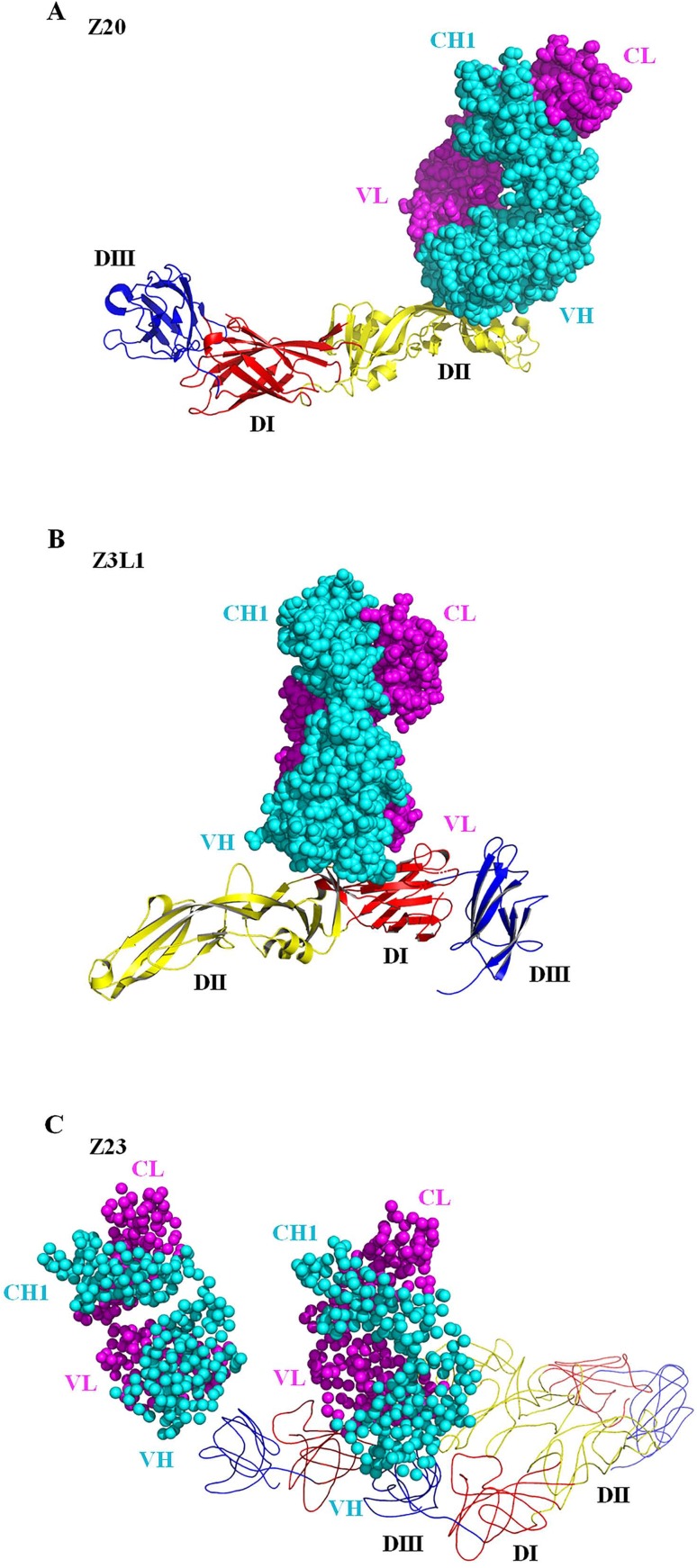
**A** The Z20 Fab binds to the central region of sE dimer from the top surface. The PDB only can output the monomeric E complexes with Fab. **B** The Z3L1 Fab binds to the distal region of the sE dimer from the top surface. The PDB only can output the monomeric E complexes with Fab. **C** The Z23 Fab binds to DIII of one envelope protein monomer and can cross-react with two envelope protein dimers on the virion surface. Due to the low resolution of complex, it could not show more details.

In another study, thirteen specific human mAbs were isolated from a single patient infected by ZIKV (Wang* et al.*^2016^). Three cryo-EM structures were obtained in complex with ZIKV E protein and ZIKV virion respectively: (1) Z20/ZIKV E (PDB: 5GZO) (Fig. 3A), (2) Z3L1/ZIKV E (PDB: 5GZN) (Fig. 3B) and (3) Z23/ZIKV (PDB: 5GZR) (Fig. 3C). For Z20/ZIKV E complex, two Z20 Fab molecules were bound to the central region of the E dimer top surface. For the Z3L1/ZIKV E complex, two Z3L1 Fabs were bound to the distal region of the E dimer top surface. For the Z23/ZIKV viron complex, it was found that 120 Z23 Fab copies were bound to one ZIKV particle. Z23 mainly binds to DIII of one envelope protein monomer and can cross-react with two envelope protein dimers on the virion surface (Wang* et al.*^2016^). Although the binding sites of these three mAbs are different, the mechanism of neutralization of the virus is similar. They can block the conformational change from dimer protein E to trimer during the membrane fusion process (Wang* et al.*^2016^).

## Prospects for Development of Neutralizing Antibodies against ZIKV Based on More Refined Structural Information

Even after all the studies that have been done to map different epitopes in ZIKV, still a lot of areas remain elusive. Structure-guided analysis, mainly using cryo-EM single particle approaches as well as computational simulations, will be very helpful for optimal epitope selection as a key step for engineering new neutralizing antibodies against the virus. Structural vaccinology, in which protein structure information is used to design immunogens, has promise to provide new vaccines against difficult targets, as ZIKV.

Within the last few years, several key advances have allowed for better and better resolution on the cryo-EM field: first, a new type of camera (direct electron detectors) has been introduced, which allows for much better signal detection; and second, more computing power coupled with new algorithms for processing images has allowed researchers to tease more information out of existing electron microscopic images. For the first time, it is possible to acquire near-atomic resolution information from cryo-EM. This technology will be key to map different epitopes for new mAbs targeting ZIKV. Our main goal will be to use this structural information to optimize a vaccine design approach.

However, electron microscopists know that single particle cryo-EM is not the only way to understand antibody–antigen interactions. We know that flexibility is not good for cryo-EM. Some antibody–antigen binding can show some degree of flexibility. In those cases, those epitopes cannot be resolved by EM. Using computational simulations as well as small angle neutron scattering (SANS) will be very helpful to resolve those flexible areas.

Our plan is to combine all these technologies: Cryo-EM, SANS as well as computational modeling to develop a better vaccine development approach against ZIKV.

## References

[CR1] Barba-Spaeth G, Dejnirattisai W, Rouvinski A, Vaney MC, Medits I, Sharma A, Simon-Loriere E, Sakuntabhai A, Cao-Lormeau VM, Haouz A, England P, Stiasny K, Mongkolsapaya J, Heinz FX, Screaton GR, Rey FA (2016). Structural basis of potent Zika-dengue virus antibody cross-neutralization. Nature.

[CR2] Dai L, Song J, Lu X, Deng YQ, Musyoki AM, Cheng H, Zhang Y, Yuan Y, Song H, Haywood J, Xiao H, Yan J, Shi Y, Qin CF, Qi J, Gao GF (2016). Structures of the Zika virus envelope protein and its complex with a flavivirus broadly protective antibody. Cell Host Microbe.

[CR3] Dejnirattisai W, Jumnainsong A, Onsirisakul N, Fitton P, Vasanawathana S, Limpitikul W, Puttikhunt C, Edwards C, Duangchinda T, Supasa S, Chawansuntati K, Malasit P, Mongkolsapaya J, Screaton G (2010). Cross-reacting antibodies enhance dengue virus infection in humans. Science.

[CR4] Dejnirattisai W, Wongwiwat W, Supasa S, Zhang X, Dai X, Rouvinski A, Jumnainsong A, Edwards C, Quyen NTH, Duangchinda T, Grimes JM, Tsai WY, Lai CY, Wang WK, Malasit P, Farrar J, Simmons CP, Zhou ZH, Rey FA, Mongkolsapaya J, Screaton GR (2015). A new class of highly potent, broadly neutralizing antibodies isolated from viremic patients infected with dengue virus. Nat Immunol.

[CR5] Della-Porta AJ, Westaway EG (1978). A multi-hit model for the neutralization of animal viruses. J Gen Virol.

[CR6] Deng YQ, Dai JX, Ji GH, Jiang T, Wang HJ, Yang HO, Tan WL, Liu R, Yu M, Ge BX, Zhu QY, Qin ED, Guo YJ, Qin CF (2011). A broadly flavivirus cross-neutralizing monoclonal antibody that recognizes a novel epitope within the fusion loop of E protein. PLoS ONE.

[CR7] Dowd KA, Pierson TC (2011). Antibody-mediated neutralization of flaviviruses: a reductionist view. Virology.

[CR8] Fernandez E, Dejnirattisai W, Cao B, Scheaffer SM, Supasa P, Wongwiwat W, Esakky P, Drury A, Mongkolsapaya J, Moley KH, Mysorekar IU, Screaton GR, Diamond MS (2017). Human antibodies to the dengue virus E-dimer epitope have therapeutic activity against Zika virus infection. Nat Immunol.

[CR9] Fibriansah G, Lok SM (2016). The development of therapeutic antibodies against dengue virus. Antivir Res.

[CR10] Gerold G, Bruening J, Weigel B, Pietschmann T (2017). Protein interactions during the flavivirus and hepacivirus life cycle. Mol Cell Proteomics.

[CR11] Harrison SC (2008). The pH sensor for flavivirus membrane fusion. J Cell Biol.

[CR12] Hasan SS, Miller A, Sapparapu G, Fernandez E, Klose T, Long F, Fokine A, Porta JC, Jiang W, Diamond MS, Crowe JE, Kuhn RJ, Rossmann MG (2017). A human antibody against Zika virus crosslinks the E protein to prevent infection. Nat Commun.

[CR13] Hasan SS, Sevvana M, Kuhn RJ, Rossmann MG (2018). Structural biology of Zika virus and other flaviviruses. Nat Struct Mol Biol.

[CR14] Kostyuchenko VA, Lim EX, Zhang S, Fibriansah G, Ng TS, Ooi JS, Shi J, Lok SM (2016). Structure of the thermally stable Zika virus. Nature.

[CR15] Li M, Wang X, Wang Q, Yu L, Wang L, Yan J, Zhang F, Zhang L, Gao GF, Jin X (2017). Both structure and function of human monoclonal antibodies contribute to enhancement of Zika virus infectivity *in vitro*. Sci China Life Sci.

[CR16] Lu LL, Suscovich TJ, Fortune SM, Alter G (2018). Beyond binding: antibody effector functions in infectious diseases. Nat Rev Immunol.

[CR17] Munjal A, Khandia R, Dhama K, Sachan S, Karthik K, Tiwari R, Malik YS, Kumar D, Singh RK, Iqbal HMN, Joshi SK (2017). Advances in developing therapies to combat Zika virus: current knowledge and future perspectives. Front Microbiol.

[CR18] Perera-Lecoin M, Meertens L, Carnec X, Amara A (2013). Flavivirus entry receptors: an update. Viruses.

[CR19] Qu P, Zhang W, Li D, Zhang C, Liu Q, Zhang X, Wang X, Dai W, Xu Y, Leng Q, Zhong J, Jin X, Huang Z (2018). Insect cell-produced recombinant protein subunit vaccines protect against Zika virus infection. Antivir Res.

[CR20] Robbiani DF, Bozzacco L, Keeffe JR, Khouri R, Olsen PC, Gazumyan A, Schaefer-Babajew D, Avila-Rios S, Nogueira L, Patel R, Azzopardi SA, Uhl LFK, Saeed M, Sevilla-Reyes EE, Agudelo M, Yao KH, Golijanin J, Gristick HB, Lee YE, Hurley A, Caskey M, Pai J, Oliveira T, Wunder EA, Sacramento G, Nery N, Orge C, Costa F, Reis MG, Thomas NM, Eisenreich T, Weinberger DM, de Almeida ARP, West AP, Rice CM, Bjorkman PJ, Reyes-Teran G, Ko AI, MacDonald MR, Nussenzweig MC (2017). Recurrent potent human neutralizing antibodies to Zika virus in Brazil and Mexico. Cell.

[CR21] Sapparapu G, Fernandez E, Kose N, Bin C, Fox JM, Bombardi RG, Zhao H, Nelson CA, Bryan AL, Barnes T, Davidson E, Mysorekar IU, Fremont DH, Doranz BJ, Diamond MS, Crowe JE (2016). Neutralizing human antibodies prevent Zika virus replication and fetal disease in mice. Nature.

[CR22] Sevvana M, Long F, Miller AS, Klose T, Buda G, Sun L, Kuhn RJ, Rossmann MG (2018). Refinement and analysis of the mature Zika virus cryo-EM structure at 3.1 Å resolution. Structure.

[CR23] Sirohi D, Kuhn RJ (2017). Zika virus structure, maturation, and receptors. J Infect Dis.

[CR24] Sirohi D, Chen Z, Sun L, Klose T, Pierson TC, Rossmann MG, Kuhn RJ (2016). The 3.8 Å resolution cryo-EM structure of Zika virus. Science.

[CR25] Stettler K, Beltramello M, Espinosa DA, Graham V, Cassotta A, Bianchi S, Vanzetta F, Minola A, Jaconi S, Mele F, Foglierini M, Pedotti M, Simonelli L, Dowall S, Atkinson B, Percivalle E, Simmons CP, Varani L, Blum J, Baldanti F, Cameroni E, Hewson R, Harris E, Lanzavecchia A, Sallusto F, Corti D (2016). Specificity, cross-reactivity, and function of antibodies elicited by Zika virus infection. Science.

[CR26] Stiasny K, Heinz FX (2006). Flavivirus membrane fusion. J Gen Virol.

[CR27] Tai W, He L, Wang Y, Sun S, Zhao G, Luo C, Li P, Zhao H, Fremont DH, Li F, Jiang S, Zhou Y, Du L (2018). Critical neutralizing fragment of Zika virus EDIII elicits cross-neutralization and protection against divergent Zika viruses. Emerg Microbes Infect.

[CR28] Wang Q, Yang H, Liu X, Dai L, Ma T, Qi J, Wong G, Peng R, Liu S, Li J, Li S, Song J, Liu J, He J, Yuan H, Xiong Y, Liao Y, Li J, Yang J, Tong Z, Griffin BD, Bi Y, Liang M, Xu X, Qin C, Cheng G, Zhang X, Wang P, Qiu X, Kobinger G, Shi Y, Yan J, Gao GF (2016). Molecular determinants of human neutralizing antibodies isolated from a patient infected with Zika virus. Sci Transl Med.

[CR29] Wang Q, Yan J, Gao GF (2017). Monoclonal antibodies against Zika virus: therapeutics and their implications for vaccine design. J Virol.

[CR30] Wu Y, Li S, Du L, Wang C, Zou P, Hong B, Yuan M, Ren X, Tai W, Kong Y, Zhou C, Lu L, Zhou X, Jiang S, Ying T (2017). Neutralization of Zika virus by germline-like human monoclonal antibodies targeting cryptic epitopes on envelope domain III. Emerg Microbes Infect.

[CR31] Zhao H, Fernandez E, Dowd KA, Speer SD, Platt DJ, Gorman MJ, Govero J, Nelson CA, Pierson TC, Diamond MS, Fremont DH (2016). Structural basis of Zika virus-specific antibody protection. Cell.

